# Radiation doses in endovascular revascularisation of lower-extremity arterial diseases

**DOI:** 10.5830/CVJA-2022-046

**Published:** 2022-09-23

**Authors:** brahim Çağrı Kaya, Fatma Altuntaş Kaya

**Affiliations:** Eskisehir City Hospital, Eskisehir, Turkey; Department of Neurology, Eskisehir Osmangazi University Medical Faculty, Eskisehir, Turkey

**Keywords:** endovascular revascularisation, peripheral arterial disease, radiation exposure

## Abstract

**Background:**

:The use of percutaneous endovascular intervention in lower-extremity arterial diseases is increasing daily. With the growing technical experience of vascular surgeons, this is preferred to open surgery in more complex lesions.

**Methods:**

The dose area product (DAP) and fluoro (FL) time values of 150 patients who underwent successful peripheral endovascular arterial intervention were analysed retrospectively. These values were evaluated by grouping according to the anatomical region and complexity of the lesion, type of procedure and arterial access.

Results: While the mean DAP was 18 ± 27 Gy cm2 in patients who underwent only angioplasty, it was 21 ± 17 Gy cm2 in patients who underwent stent implantation after angioplasty (p = 0.069). The DAP value was statistically significantly higher in patients who had intervention in the pelvic region, both in the angioplasty (23 ± 22 Gy cm2) group and in the stenting (29 ± 18 Gy cm2) group, than in patients who had intervention in the femoropopliteal region (18 ± 27 and 15 ± 12 Gy cm2, respectively) (p < 0.05). When the correlation between body mass index (BMI) of the patients and DAP was examined, a moderate positive correlation was found both in the pelvic region (r = 0.601, p = 0.00) and in the femoropopliteal region (r = 0.512, p = 0.00). Out of 78 patients in whom the ipsilateral popliteal retrograde approach was preferred, only two developed arteriovenous fistulae after the procedure, and only two of 77 patients in whom the femoral approach was preferred developed no major or minor complications, except femoral pseudo-aneurysm.

**Conclusions:**

The most important factors affecting the radiation doses of the patients were the anatomical region and the patient’s BMI. Radiation doses were higher in pelvic interventions compared to the femoropopliteal region. This may encourage the choice of arterial approaches that can minimise visualisation of the pelvic region in particular. Therefore, attention should be paid to pre-operative planning, especially in patients undergoing multiple diagnostic and therapeutic imaging. The ipsilateral popliteal retrograde approach can be safely chosen in combined iliofemoral, common femoral and superficial femoral total occlusions in the hands of surgeons with good Doppler ultrasonography experience.

Percutaneous endovascular revascularisation interventions are widely used in increasingly complex cases of lower-extremity arterial disease (LEAD). Particularly in our country, the increasing experience and success rates over time of vascular surgeons in endovascular interventions have led to fewer open surgical procedures for the lower extremities. This has increased the number of studies conducted, which report fewer complications, lower radiation doses and higher success rates.^1^

Factors such as the complexity of the lesion, its anatomical location and vascular structure can affect selection of the arterial access site to reach the lesion. Patient and operator radiation doses are undoubtedly very important in this selection. Monitoring patients’ exposure to X-rays is crucial to minimising the harmful effects of radiation and is an integral part of monitoring the quality of work in interventional units.^2^

The values most commonly used in studies of patients’ radiation doses are fluoroscopy (FL) time and dose area product (DAP), defined as the radiation dose absorbed multiplied by the irradiated area and expressed as gray square centimetres (Gy cm^2^). It has been suggested that DAP is the most effective value in predicting the risk of malignancy due to radiation, therefore it can be used to predict harmful effects. It can also be used to compare doses between different types of procedures in the same anatomical region and between the same procedures in different centres.^3^

The aim of this study was to retrospectively evaluate the radiation exposure of patients during endovascular revascularisation in a single tertiary medical centre, according to the anatomical region, type of intervention, procedure performed and the complexity of arterial lesions.

## Methods

We retrospectively analysed the data regarding peripheral endovascular revascularisation interventions performed between December 2018 and November 2020, in patients with symptomatic peripheral arterial disease, intermittent claudication and critical leg ischaemia, by the cardiovascular surgery clinic of Eskişehir City Hospital. While patients who underwent arterial revascularisation were included in the study, patients whose arterial system could not be accessed or the lesion could not be passed were excluded from the study.

Patients’ data were retrieved from the electronic hospital medical record system, the imaging archiving and communication system and the angiography X-ray system. Since this was a retrospective, descriptive study of standard treatment procedures using anonymised data, no ethical approval of the institutional ethics board was needed.

In all patients, the diagnosis of LEAD was set clinically and confirmed with the measurement of ankle-brachial pressure index (ABI). The Fontaine stage of LEAD was recorded, namely, having disabling claudication, rest pain, ulceration or gangrene. Risk factors for LEAD were recorded, including arterial hypertension, defined as arterial blood pressure > 140/90 mmHg or using antihypertensive medication; hypercholesterolaemia, defined as having serum low-density lipoprotein cholesterol ≥ 130 mg/dl (3.37 mmol/l) or total cholesterol ≥ 200 mg/dl (5.18 mmol/l) or using lipolytic medication; diabetes, defined as having a fasting blood glucose level ≥ 126 mg/dl (6.99 mmol/l) or using antidiabetic medication; and self-reported past or current smoking.

Prior to the endovascular procedure, computed tomographic angiography (CTA) was performed in all patients so that the most appropriate approach for the arterial system was chosen. Principles of endovascular treatment of symptomatic LEAD, as described in the European Society of Cardiology guidelines, were followed.^4^

Interventions were performed by two different interventionists in one angiography laboratory, using a Canon Infinix-I INFX 8000-V single-plane Toshiba angiography system on a movable interventional table. Patients in a supine or prone position according to the puncture point, were placed into the appropriate field of view by a manually controlled sliding table. A posterior– anterior projection was routinely used. An oblique projection was used occasionally, to confirm the presence of stenosis and/ or evaluate the result of angioplasty. Magnification was used only occasionally, mostly in below-knee interventions, at the discretion of the interventionist.

A 6F sheath was used in all therapeutic interventions. In the therapeutic interventions with a retrograde approach, digital subtraction angiography (DSA) was performed through a pigtail catheter. Iohexol contrast medium of 350 mg/ml (Omnipaque 350, GE HealthCare, Ireland) was manually injected into the arterial system by the interventionist. On average, 100 mm^3^ (175 mg/ml) contrast medium was used for the patients who underwent aorto-iliac therapeutic interventions.

When revascularisation was judged technically not possible, the intervention was completed following DSA as a diagnostic intervention and these patients were excluded from the study. In all other patients, an attempt at endovascular revascularisation was made and the intervention was categorised as a therapeutic intervention. Therapeutic interventions were defined as technically successful when recanalisation of the arterial lumen with a less than 30% residual stenosis and rapid contrast flow was achieved.

In patients with combined iliofemoral lesions, the ipsilateral popliteal retrograde approach was performed with Doppler ultrasonography in the prone position. In therapeutic interventions, angioplasty was performed primarily on the lesion. In control imaging, if dissection was detected, bailout stenting was performed.

Therapeutic interventions in the femoropopliteal region were generally performed with an antegrade ipsilateral approach. When recanalisation of the femoral artery was not successful (no re-entry), a retrograde ipsilateral–popliteal approach was performed.

For analysis, interventions were categorised according to the following criteria:

- anatomical region (pelvic, femoropopliteal ± below knee)
- type of the intervention (angioplasty without stenting, angioplasty with stent implantation)
- puncture point for approaching the arterial lesions.

The patients were divided into groups according to their clinical status before the procedure, using the Fontaine classification. The DAP and FL time were recorded by the system DAP meter, which was an integral part of the angiography equipment.

## Statistical analysis

All statistical analyses were made with the IBM SPSS Statistic version 23.0 for Windows. Data were tested in terms of normal distribution with the Kolmogorov–Smirnov test. DAP and FL time values with regard to the anatomical region were tested with the Mann–Whitney U-test due to continuous data without normal distribution. Kruskal–Wallis and Tukey tests were used for comparisons with regard to puncture point. The Spearman correlation test was used to measure the change in the values according to age and body mass index (BMI).

## Results

Therapeutic peripheral endovascular arterial intervention was performed in 150 patients over a period of 23 months. The basic characteristics of the patients are shown in Table 1. All patients included in the study had ABI values before and after the procedure. It was observed that the mean ABI, which was 0.58 ± 0.17 before the procedure, increased to 0.93 ± 0.19 after the procedure.

**Table 1:** Patient characteristics

*Variables*	*Values*
Gender: male/female, n (%)	129/21 (86/14)
Age, mean + SD (min-max)	62 + 8 (43-77)
Hypertension, n (%)	72 (48)
Hypercholesterolaemia, n (%)	63 (42)
Diabetes mellitus, n (%)	81 (54)
Current or former smoker, n (%)	108 (72)
Body mass index (kg/m²), mean + SD (min-max)	27 + 4 (20-36)
Bilateral extremity lesions, n (%)	33 (22)
Angioplasty/angioplasty with stenting, n (%)	99/51 (66/34)
Pre-op ABI, mean + SD (min-max)	0.58 + 0.17 (0.2-0.89)
Postop ABI, mean + SD (min-max)	0.93 + 0.19 (0.43-1.3)
Fluoro time (min)	11.6 + 8.4 (3-39)
	(Op 1: 10.3 and Op 2: 12.7)
DAP (Gycm2)	19.5 + 24 (2-130) (Op1 18.2 and Op 2: 20.8)
Fluoro time (min), median (IQR)	8.5 (9)
DAP (Gycm2), median (IQR)	11 (20)

SD: standard deviation, ABI: ankle-brachial index, DAP: dose area product,

Op: operator.

While evaluating our results, the mean DAP and FL time values were compared to show that there was no operator effect, and it was proved that there was no statistically significant difference (p = 0.042 for DAP, p = 0.066 for Fl time). The FL time and DAP values of the patients who underwent revascularisation with only balloon angioplasty and those who underwent stent implantation upon development of dissection during the procedure were higher in the stent-implanted group (Table 2). For this reason, both groups were evaluated separately in further analysis.

**Table 2:** Comparison of patients who underwent angioplasty and those who underwent stenting after angioplasty

*Variables*	*Angioplasty (mean + SD)*	*Angioplasty with stent*	p-value
FL time (min)	10 + 8	15 + 9	0.00
DAP (Gy cm2)	18 + 27	21 + 17	0.069

No patient developed radiation damage to the skin. An arteriovenous fistula was detected after the procedure in only two of the 78 patients in whom the ipsilateral popliteal retrograde approach in the prone position was preferred. The fistula was closed in both patients with balloon angioplasty and their follow up was normal. Pseudo-aneurysm developed in only two of 77 patients in whom the femoral approach was preferred and no surgical operation was required in these two patients. No closure device was used in any patient.

The data according to the anatomical region of the arterial lesion were compared with the Mann–Whitney U-test after being subjected to a distribution test with the Kolmogorov–Smirnov test. These values are given in Table 3. It was observed that the DAP value of the pelvic region was higher in both groups.

**Table 3:** Comparison of patients according to the anatomical region of the arterial lesion

				*p-value*	
*Angioplasty*	*No. FL*	*time (min)*	*DAP (Gycm²)*	*FL time*	*DAP*
Angioplasty without stenting					
Pelvic region	15	7 1 - 1	23 + 22	0.215	0.020
Femoropopliteal region	84	11 + 8	18 + 27		
Angioplasty with stenting					
Pelvic region	24	18 + 9	29 + 18	0.014	0.004
Femoropopliteal region	27	12 + 7	15 + 12		

When the patients with aorto-iliac lesions were divided into groups according to the Fontaine classification, no difference was found between the subgroups (Table 4), (FL time p = 0.137, DAP p = 0.181).

**Table 4:** Comparison of patients according to Fontaine classification

*Fontaine classification*	*Number (%)*	*FL time (min)*	*DAP (Gycm²)*
2A	51 (34)	12 + 10	20 + 29
2B	63 (42)	12 + 8	16 + 15
3	30 (20)	12 + 7	26 + 29
4	6 (4)	6 + 1	22 + 3

Arterial intervention was achieved with four different approaches, planned before the procedure according to the pre-operative CTA images of our patients. Particularly in combined iliofemoral lesions and long-segment chronic femoral total occlusions, the patient was placed in the prone position and the ipsilateral popliteal retrograde approach was preferred with Doppler ultrasound.

DAP and FL time values according to arterial puncture point are shown in Table 5. It was shown that these values were higher in patients with ipsilateral popliteal retrograde puncture than in patients with ipsilateral femoral antegrade puncture (FL time p = 0.00, DAP p = 0.005)

**Table 5:** Comparison of patients according to arterial access

*Puncture point*	*Number (%)*	*FL time (min)*	*DAP (Gycm²)*
Ipsilateral popliteal retrograde	78 (52)	13 + 9	25 + 29
Ipsilateral femoral retrograde	15 (10)	9 + 2	21 + 24
Contralateral femoral retrograde	27 (18)	15 + 8	15 + 8
Ipsilateral femoral antegrade	30 (20)	5 + 3	8 + 11

**Fig. 1:**
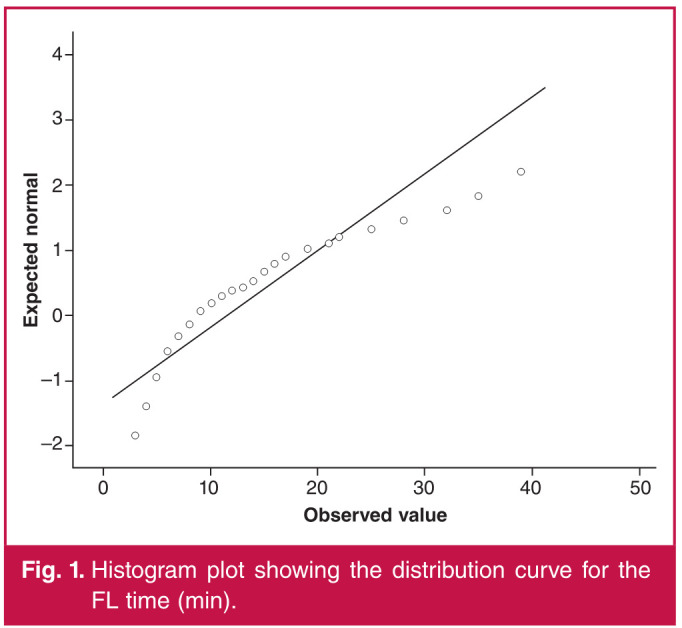
Histogram plot showing the distribution curve for the FL time (min).

**Fig. 2:**
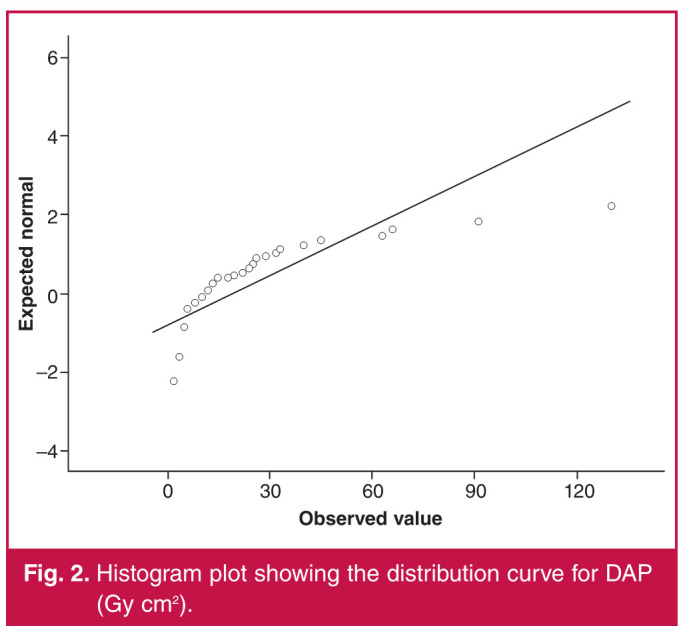
Histogram plot showing the distribution curve for DAP (Gy cm^2^).

**Fig. 3:**
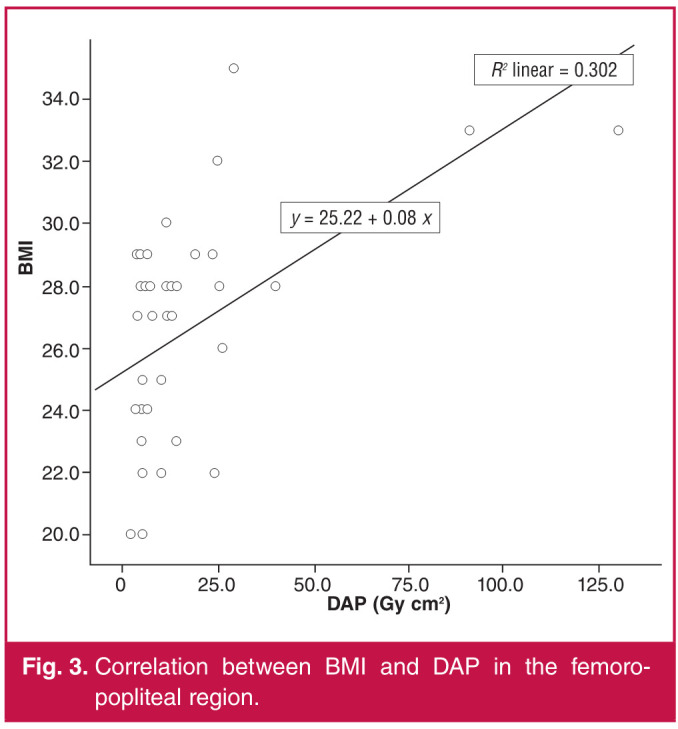
Correlation between BMI and DAP in the femoropopliteal region.

**Fig. 4:**
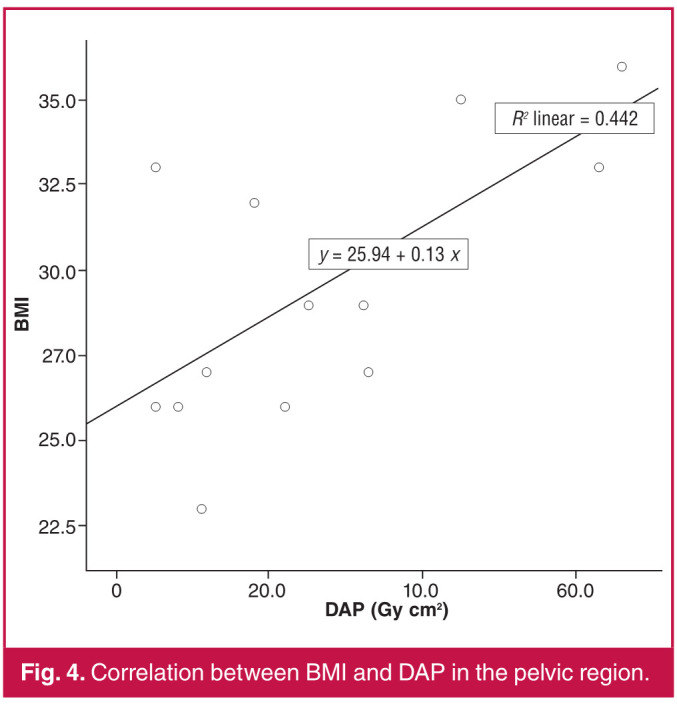
Correlation between BMI and DAP in the pelvic region.

The Spearman correlation test was used for comparisons between age and BMI values of the patients and DAP and FL time. A weak negative correlation was found between the age of our patients and their BMI (r = –0.270, p = 0.001). There was a moderate positive correlation between DAP in patients with both pelvic (r = 0.601, p = 0.00) and femoropopliteal region (r = 0.512, p = 0.00) interventions (Figs 1, 2) and BMI. There was no significant correlation between FL time and BMI (p = 0.544, p = 0.124). While there was no correlation between age and DAP in the femoropopliteal region (p = 693), a moderate negative correlation (r = –0.493, p = 0.001) was found between DAP in the pelvic region and BMI (Figs 3, 4).

## Discussion

In this study, we investigated how the anatomical region of the arterial lesion, the complexity of the lesion, the patient’s BMI, age, type of endovascular intervention and preferred puncture point to reach the lesion affected the patient’s radiation dose. The most important factors affecting the radiation dose were the anatomical region, the patient’s BMI and the type of endovascular intervention. Pelvic interventions for aorta-iliac lesions had higher DAP values than interventions below the iliac ligament that included femoropopliteal lesions.

Regarding high radiation doses in pelvic interventions, similar results were reported in studies by Boc et al.^5^ and Sigterman et al.^6^ The main reason for this is explained as follows: the mass and properties of the tissues exposed to radiation in the pelvic region direct the automatic exposure control of the X-ray system, which aims to keep the image brightness and contrast constant, to higher tube current and voltage and consequently to higher dose rates.^7^ The positive correlation between BMI values of our patients and DAP is consistent with this explanation (Figs 1, 2).

Behrendt et al. reported that female and elderly patients had lower DAP values than males and younger patients.^8^ Considering that we also found a negative correlation between the ages of our patients and DAP values, it seemed to be consistent with our results.

High radiation rates in the pelvic region, mainly due to tissue properties, require the avoidance of pelvic region radiation when choosing the approach preferences, especially in the revascularisation of femoropopliteal lesions. Using the contralateral approach only when there are valid reasons against the ipsilateral approach should be encouraged in view of the ‘as low as reasonably achievable (ALARA)’ principles.^2^

In our patients, we preferred the ipsilateral popliteal retrograde approach rather than the contralateral femoral retrograde approach in combined iliofemoral and long-segment total occlusions. Disadvantages of this method such as long stay in the prone position, development of popliteal pseudoaneurysm and arteriovenous fistula were mentioned.^10^ However, arteriovenous fistulae developed in only two of 78 patients in our series, and surgical operation was not required in these patients. There has been an increasing number of studies advocating that this method can be performed safely and effectively in the hands of operators with good Doppler ultrasonography experience.^9^,^11^

When the radiation doses were examined, the DAP value of the patients in whom we preferred the ipsilateral popliteal retrograde approach was found to be statistically higher than those in whom we preferred only the ipsilateral femoral antegrade approach. Considering that we mostly prefer the ipsilateral popliteal retrograde approach in patients with lesions of the common femoral artery, iliac artery or even bilateral lesions where ipsilateral femoral puncture is not possible, it can be concluded that our preference is safe in terms of radiation doses.

## Limitations

The main limitation of our study was that it was a retrospective, single-centre study. Our number of contralateral femoral retrograde approaches could have been higher, but we believe that our ipsilateral femoral retrograde approach preference standards and the high success and low complication rates in the procedure affected this. The relatively low percentage of complex lesions could be attributed to the low DAP values in our study. Our angiographic equipment did not provide information about air kerma to the interventional reference point and we did not use this skin dose in our analysis.

## Conclusions

The incidence of endovascular interventions for lower-extremity arteries are growing daily, and patient and operator radiation doses are increasing as more complex lesions are intervened. Therefore, attention should be paid to pre-operative planning, especially in patients undergoing multiple diagnostic andtherapeutic imagings. Various clinical factors, often known prior to an intervention, such as the location of the disease and the intended access route, can help predict and limit the radiation dose. This information may also help determine the mode of intervention or encourage alternative therapies. The most important factors affecting radiation doses of the patient are the anatomical region and the patient’s BMI. Radiation doses are higher in pelvic interventions, which may be an incentive to prefer arterial approaches that can minimise imaging of the pelvic area. We prefer the ipsilateral popliteal retrograde approach with Doppler ultrasound with the patient in the prone position as it is a safe and effective method with low complication and high success rates. 
